# The Effect of a Short-Term Exposure to Lead on the Levels of Essential Metal Ions, Selected Proteins Related to Them, and Oxidative Stress Parameters in Humans

**DOI:** 10.1155/2017/8763793

**Published:** 2017-12-13

**Authors:** Michał Dobrakowski, Marta Boroń, Ewa Birkner, Aleksandra Kasperczyk, Ewa Chwalińska, Grażyna Lisowska, Sławomir Kasperczyk

**Affiliations:** ^1^Department of Biochemistry, School of Medicine with the Division of Dentistry, Medical University of Silesia, Ul. Jordana 19, 41-808 Zabrze, Poland; ^2^Institute of Occupational Medicine and Environmental Health in Sosnowiec, Ul. Kościelna 13, 41-200 Sosnowiec, Poland; ^3^Department of Otorhinolaryngology and Laryngological Oncology, School of Medicine with the Division of Dentistry, Medical University of Silesia, Ul. Skłodowskiej 10, 41-800 Zabrze, Poland

## Abstract

The present study was designed to explore the possible influence of subacute exposure to lead on the levels of selected essential metals, selected proteins related to them, and oxidative stress parameters in occupationally exposed workers. The study population included 36 males occupationally exposed to lead for 36 to 44 days. Their blood lead level at the beginning of the study was 10.7 ± 7.67 *μ*g/dl and increased to the level of 49.1 ± 14.1 *μ*g/dl at the end of the study. The levels of calcium, magnesium, and zinc increased significantly after lead exposure compared to baseline by 3%, 3%, and 8%, respectively, while the level of copper decreased significantly by 7%. The malondialdehyde (MDA) level and the activities of catalase (CAT) and superoxide dismutase (SOD) did not change due to lead exposure. However, the level of lipid hydroperoxides (LPH) in serum increased significantly by 46%, while the level of erythrocyte lipofuscin (LPS) decreased by 13%. The serum levels of essential metals are modified by a short-term exposure to lead in occupationally exposed workers. A short-term exposure to lead induces oxidative stress associated with elevated levels of LPH but not MDA.

## 1. Introduction

Lead is one of the ubiquitous pollutants and exposure to it is a global concern. Lead is commonly used in many industries, and its disposal has resulted in its accumulation in the environment [1, 2]. There is no known physiological value of lead, and there is no safe level of exposure to this xenobiotic. Even low blood lead levels (<10 *μ*g/dl) are believed to be associated with abnormalities in hematopoietic, nervous, and renal systems [3].

Occupational lead exposure occurs mainly through the respiratory tract. Approximately 30–40% of inhaled lead is absorbed into the bloodstream. 99% of circulating lead is bound to erythrocytes for approximately 30–35 days and is distributed into the tissues, such as the liver, renal cortex, blood vessels, brain, lungs, spleen, teeth, and bones, over the following 4–6 weeks. In adults, approximately 80–95% of absorbed lead is stored in bones [4].

There are many proposed mechanisms of toxic lead action. It has been established that lead induces oxidative stress by generation of reactive oxygen species (ROS) and impairment of antioxidant defenses, including enzymatic antioxidants, such as superoxide dismutase and catalase [5]. Besides, lead interferes with divalent cations, such as calcium (Ca), magnesium (Mg), iron (Fe), zinc (Zn), copper (Cu), and selenium (Se) [3, 6].

Essential metals are coactivators of several important enzymes, including antioxidant enzymes, and proteins which are necessary for health maintenance [6, 7]. Therefore, their interactions with lead may affect various fundamental biological processes, including intra- and intercellular signaling, cell adhesion, protein folding and maturation, apoptosis, ionic transportation, enzyme regulation, and release of neurotransmitters [3]. On the other hand, micronutrients could influence lead's absorption, postexposure distribution, deposition, and excretion processes [1]. For example, it has been reported that a low calcium and iron diet results in increased susceptibility to lead toxicity by its increased absorption. Conversely, supplementation with zinc and iron has been shown to reduce intestinal absorption of lead [2]. Consequently, it is postulated that there are possible feedback loops between blood lead and essential metals [7].

The associations between concentration of lead and essential metals in the blood and oxidative stress intensity are not fully understood. Previous studies on this topic conducted on humans focused on occupational and environmental chronic lead exposure [6]. In this context, investigation of the effects of a short-term exposure to lead would provide more information. Therefore, the aim of the present study was to explore the possible influence of subacute exposure to lead on the levels of selected micro and macro elements, selected proteins related to them, and oxidative stress parameters in occupationally exposed workers.

## 2. Material and Methods

### 2.1. Study Population

The experimental setup has been approved by the Bioethics Committee of the Medical University of Silesia in Katowice No. KNW/0022/KB1/108/14.

The study population included 36 males occupationally exposed to lead for 36 to 44 days. All participants were recruited by an occupational medicine specialist during prophylactic medical examinations and provided informed consent to the study. Blood lead level (B-Pb) served as an exposure marker. Half of the workers were only environmentally exposed to lead before the study, while the second half had a history of an occupational exposure to lead. They had been exposed to lead several years before the study began. Periods of lead exposures in the past lasted for several months to several years. Additively, the exposed population was divided into pairs of subgroups based on smoking habits and a median of age and BMI. In the examined group, 5% and 3% of workers were diagnosed with hypertension and coronary artery disease, respectively, while none of them were diagnosed with diabetes and malignant neoplasm.

Subjects in the examined group worked in lead-zinc works in the southern region of Poland to perform periodic maintenance of blast furnaces and production lines. The mean concentration of lead in the air at the participants' workplaces was 0.083 ± 0.12 mg/m^3^. This mean concentration exceeds the Polish MAC level of 0.050 mg/m^3^. Besides, examined subjects were exposed to negligible doses of zinc. The mean concentration of zinc in the air at the participants' workplaces was 0.15 ± 0.13 mg/m^3^. This value is much lower than the Polish MAC level of 5.0 mg/m^3^.

Blood of all examined workers was drawn during prophylactic medical examinations at the beginning of the studied lead exposure for the first time and after a period of short-term exposure to lead for the second time. In the collected blood samples, the biochemical analysis included the activities of superoxide dismutase (SOD) and catalase (CAT) and the levels of essential metals, ceruloplasmin (CER), lipid hydroperoxides (LPH), malondialdehyde (MDA), and lipofuscin (LPS).

This study was supported by the Medical University of Silesia in Katowice (KNW-1-063/N/6/O).

### 2.2. Laboratory Procedures

#### 2.2.1. Blood Collection

We followed the methods of Dobrakowski et al. [8]. To obtain whole blood, erythrocytes, and leukocytes, 14 ml of blood was drawn by venipuncture into tubes containing an EDTA solution as an anticoagulant. Immediately after blood sampling, 5 ml of whole blood was centrifuged. The plasma supernatant was removed. The sedimented erythrocytes were washed three times through centrifugation with 0.9% sodium chloride solution. Subsequently, the erythrocytes were lysed with bidistilled water. Finally, 10% (*v*/*v*) hemolysate was prepared. To isolate the leukocytes, 3 ml of the whole blood was layered over Histopaque-1077 (Sigma-Aldrich) in a 1 : 1 ratio and centrifuged for 30 min. Leukocytes (1.5 ml) were collected from the interface and washed three times through centrifugation with 0.9% sodium chloride solution. Finally, the lysate of leukocytes was prepared in 1.5 ml of bidistilled water.

#### 2.2.2. Determination of Lead and Essential Metal Concentrations

The assessments of the PbB (wavelength 283.3 nm) and serum concentration of selenium (196.0 nm) were performed by graphite furnace atomic absorption spectrometry using an ICE 3400 instrument (Thermo Fisher Scientific, Waltham, MA, USA). The detection limits for PbB were 0.08 *μ*g/dl and for Se 0.32 *μ*g/dl. Data were shown in micrograms per deciliter (*μ*g/dl). The assessments of the serum concentrations of magnesium (wavelength 285.2 nm), calcium (422.7 nm), iron (248.3 nm), zinc (213.9 nm), and copper (324.8 nm) were performed by air-acetylene flame atomic absorption spectrometry using an ICE 3300 instrument (Thermo Fisher Scientific, Waltham, MA, USA). The detection limits for Mg were 0.0022 mg/dl, for Ca 0.0037 mg/dl, for Fe 0.0043 mg/dl, for Zn 0.0033 mg/dl, and Cu 0.0045 mg/dl. Concentrations of Ca and Mg were shown in milligrams per deciliter (mg/dl); Fe, Zn, and Cu were shown in micrograms per deciliter (*μ*g/dl). Coefficient of variation (CV) ranged 1.64–2.18%. The overall methods recovery were 95–105%. The laboratory met the requirements of proficiency tests (Lead and Multi-Element Proficiency–CDC in Atlanta). The ClinCal® Whole Blood Calibrator and ClinCal Serum Calibrator (Recipe, Germany) were used for calibration of the instrument and control materials. ClinCheck Whole Blood Control Levels I, II, and III and ClinCheck Serum Control Levels I and II were used for quality control.

#### 2.2.3. Determination of Superoxide Dismutase (SOD) Activity

The method of Oyanagui [9] was used to measure the activity of SOD in serum, erythrocytes, and leukocytes. In this method, xanthine oxidase produces superoxide anions which react with hydroxylamine forming nitric ions. These ions react with naphthalene diamine and sulfanilic acid generating a colored product. Concentration of this product is proportional to the amount of produced superoxide anions and negatively proportional to the activity of SOD. Absorbance was measured using an automated analyzer PerkinElmer at a wavelength of 550 nm. The enzymatic activity of SOD was expressed in nitric units. The isoenzymes of SOD, such as Mn-SOD and CuZn-SOD, were also indicated in leukocytes, using KCN as the inhibitor of the CuZn-SOD activity. The activity of SOD is equal to 1 nitric unit (NU) when it inhibits nitric ion production by 50%. Activity of SOD was expressed in NU/ml in serum, NU/mg of hemoglobin (Hb) in erythrocytes, and in NU/mg of protein (P) in leukocytes.

#### 2.2.4. Determination of Catalase (CAT) Activity

Catalase activity in erythrocytes and leukocytes was measured by the method of Johansson and Borg [10] using an automated analyzer PerkinElmer. The method is based on the reaction of the enzyme with methanol in the presence of optimal concentrations of hydrogen peroxide. Formaldehyde produced is measured spectrophotometrically at 550 nm as a dye purpald. The activity of Cat-Px was expressed as U/mg P in leukocytes and as kU/g Hb in erythrocytes.

#### 2.2.5. Determination of Ceruloplasmin (CER) Concentration

The CER concentration in serum was determined spectrophotometrically by Richterich [11], using the reaction with p-phenyl diamine. To 20 ml of serum (examined sample) and to the mixture of 20 ml of serum and 200 ml of sodium azide solution (control sample), 1 ml of p-phenylenediamine dihydrochloride in acetate buffer was added. After 15 minutes of incubation, to the examined sample, 200 ml of sodium azide solution was also added. After another 15 minutes of incubation, the absorbance was measured at a wavelength of 546 nm. The values shown were in mg/dl.

#### 2.2.6. Determination of Lipid Hydroperoxide (LPH) Concentration

The concentration of lipid hydroperoxides in serum was assessed according to a method described by Södergren et al. [12]. This assay is based on the ferrous oxidation of xylenol orange following the addition of methanol and butylated hydroxytoluene (BHT). The presence of ferric ions induces a color change in xylenol orange that can be measured as the change in absorbance at 560 nm relative to blank samples containing triphenylphosphine in methanol. The assay was conducted in the Victor X3 (PerkinElmer, Waltham, MA, USA) automated analyzer, which was calibrated with hydrogen peroxide. The values corresponding to total lipid hydroperoxides were shown in *μ*mol/l.

#### 2.2.7. Determination of Malondialdehyde (MDA) Concentration

Malondialdehyde level was measured fluorometrically as a 2-thiobarbituric acid-reactive substance (TBARS) in serum according to Ohkawa et al. [13] with modifications. Samples were mixed with 8.1% sodium dodecyl sulfate, 20% acetic acid, and 0.8% 2-thiobarbituric acid. After vortexing, samples were incubated for 1 hour in 950°C and butanol-pyridine 15 : 1 (*v*/*v*) was added. The mixture was shaken for 10 minutes and then centrifuged. The butanol-pyridine layer was measured fluorometrically at 552 nm and 515 nm excitation (PerkinElmer, USA). TBARS values are expressed as malondialdehyde (MDA) equivalents. Tetraethoxypropane was used as the standard. Concentrations were given in *μ*mol/l.

#### 2.2.8. Determination of Lipofuscin (LPS) Concentration

The concentration of LPS was determined in serum and erythrocytes according to the method of Jain [14]. To the hemolysate, isopropanol and chloroform in a ratio of 3 : 2 (*v*/*v*) was added. Then, the sample was shaken for 2 minutes and after 30 minutes of incubation at 20°C was centrifuged. Fluorescence was measured in the clear supernatant using an LS45 spectrofluorometer PerkinElmer at a wavelength of 400 nm (absorbance) and 455 nm (emission). The values were presented in relative units (RU). The value of RU 100 corresponds to the fluorescence of a 0.1 mg/ml quinidine sulfate solution in sulfuric acid. The concentration of LPS was shown in RU/g Hb in erythrocytes and in RU/ml in serum.

#### 2.2.9. Statistical Analysis

The statistical analysis was performed using the Statistica 9.1 PL software program. The statistical analyses included the means and standard deviations of the data. Shapiro-Wilk test was used to verify normality, and Levene's test was used to verify the homogeneity of variances. Statistical comparisons were made using the *t*-test, *t*-test with separate variance estimates, the Mann–Whitney *U* test, or the chi-squared test. The Spearman nonparametric correlation was calculated. A value of *p* < 0.05 was considered to be significant.

## 3. Results

The mean age of the exposed population was 40.94 ± 13.68 years. Their PbB at the beginning of the study was 10.7 ± 7.67 *μ*g/dl and increased to the level of 49.1 ± 14.1 *μ*g/dl at the end of the study period (Table 1).

The levels of calcium, magnesium, and zinc increased significantly after lead exposure compared to baseline by 3%, 3%, and 8%, respectively, while the level of copper decreased significantly by 7% (Table 2).

Activities of CAT, SOD, and SOD isoenzymes did not change due to lead exposure. Analogically, the level of MDA, CER, and serum LPS did not differ when compared its values after lead exposure and at baseline. However, the level of serum LPH increased significantly by 46%, while the level of erythrocyte LPS decreased by 13% (Table 3).

The analysis of correlations showed negative correlations between the change of PbB level and changes of leukocyte SOD (*R* = −0.58) and Mn-SOD (*R* = −0.55) activities. Inverse correlations were also observed between the change of CuZn-SOD activity and the changes of MDA (*R* = −0.34) and LPH (*R* = −0.37) levels due to the studied lead exposure. Analogical correlation was found for leukocyte SOD and LPH (*R* = −0.46). Positive correlations were shown between the changes of LPH and MDA levels (*R* = 0.78) and between the change of serum LPS level and changes of the MDA (*R* = 0.47) and LPH levels (*R* = 0.42) (Table 4).

Comparisons between subgroups of workers exposed to lead before the study and workers without such exposure, smokers and non-smokers, younger and older workers, and workers with higher and lower BMI are presented in corresponding tables. Data presented in those tables include epidemiological data and parameters which are significantly different (Tables 5–8).

## 4. Discussion

Blood lead level increased five times after examined exposure period. Observed elevations of blood lead levels were caused by inadequate adherence of workers to work safety procedures. Workers were in conditions of exposure to lead for 12 hours a day. They did not properly use the personal protective equipment, such as protective clothes, masks, and goggles, probably because they neglected or were unaware of possible adverse health effects of lead poisoning. The main source of lead in the working environment is the manufacturing process of melting raw materials which releases lead to the atmosphere around the steelwork's furnace as industrial dust. Wind-blown historical dust and slag storage piles are the secondary sources of lead exposure [15].

Calcium regulates many different cellular functions, such as contraction, secretion, metabolism, gene expression, cell survival, and death. Due to the interaction with stereospecific sites for divalent cations, lead may potentially impair all of the calcium-dependent processes [16]. In the present study, serum calcium level significantly increased after a short-term lead exposure. This increase may be due to the competitive displacement of calcium from its binding sites in cells by lead. In lead-exposed individuals, the concentration of lead is 90 times higher in red blood cells than in the plasma [17]. Lead ion is transported into the erythrocytes through calcium transport systems and may compete with calcium ions [18]. In consequence, calcium uptake by erythrocytes may decrease and result in elevated calcium level in serum which was observed in the present study. On the other hand, lead is also believed to trigger opposite mechanisms leading to the increase of calcium level in red blood cells. It has been proposed that lead-induced oxidative stress may induce calcium influx by ROS-mediated activation of calcium channels and suppression of (Ca^2+^, Mg^2+^)-ATPase activity which is responsible for calcium efflux. Besides, lead ions directly inhibit (Ca^2+^, Mg^2+^)-ATPase activity [16, 17]. In cardiomyocytes, lead has been shown to activate the cascade of Src and ERK 1/2 which activates the *α*-1 subunit of L-type calcium channels. This activation results in increased calcium influx [19]. The same mechanism may operate in red blood cells. The abovementioned mechanisms explain increased calcium level reported in erythrocytes of workers chronically exposed to lead [16] and in human erythrocytes exposed to lead in vitro [17]. In accordance, a negative correlation (*R* = −0.41, *p* < 0.01) between blood lead level and plasma calcium level was shown in workers chronically exposed to lead [20]. This observation may be also due to the inhibitory effect of lead on 1*α*-hydroxylase in renal tubules. As a result, lead inhibits synthesis of calcitriol resulting in a decrease in calcium absorption in the intestine and its reabsorption in renal tubules [21]. The complexity of mentioned interactions between lead and calcium may result in divergent results of studies which are methodologically different. Besides, the analysis of confounders showed that smoking habits affect the influence of lead exposure on serum calcium level. In a group of nonsmokers, the increase of serum calcium level due to the exposure was significantly higher than in a group of smokers.

The interactions between magnesium and lead are less studied. Magnesium is known to be a calcium antagonist and also serves as an essential element for cell functioning being a cofactor in more than 300 enzymatic reactions. Protein and DNA biosynthesis, anaerobic energy production, and the hydrolysis and transfer of phosphate groups are believed to be the most important biochemical processes that require magnesium ions [22]. Analogically to calcium levels, serum magnesium levels significantly increased after a short-term exposure to lead compared to baseline. This elevation could be also caused by the increased release of magnesium from tissues due to its displacement from binding sites by lead ions. Consistently, Chiba et al. [23] found decreased magnesium level in erythrocytes of workers chronically exposed to lead (PbB = 32.52 ± 9.49 *μ*g/dl) compared to unexposed control group, while plasma magnesium level in this group of workers was the same as in the control group. However, a positive association between blood lead and serum magnesium levels was shown in a group of children environmentally exposed to high doses of lead (PbB = 19.9 ± 17.93 *μ*g/dl) [2].

Selenium is the next metal that competes with lead. Selenium is involved in the antioxidant defense as a cofactor for glutathione peroxidase (GPx) and plays a role in tissue respiration. Selenoproteins also have the antioxidant properties and help to eliminate reactive oxygen species overproduced by the metals' action. Some animal studies have shown that lead-induced oxidative stress could be reduced by the administration of selenium alone or the combination of selenium and other antioxidants [24]. In our previous study, we showed decreased level of selenium in workers chronically exposed to medium and high doses of lead compared to the unexposed control group [6]. Results of the present study indicate that a short-term exposure to lead does not significantly affect serum selenium level. However, selenium level was significantly higher after the studied period of lead exposure in a group of workers who had not a history of occupational exposure to lead than in workers with such a history. Analogical difference in selenium level due to exposure was observed between subgroups of workers with low and high BMI. These observations suggest an existence of a defense mechanism against lead toxicity which is efficient in individuals with lower BMI and without a history of occupational lead exposure.

Similarly to selenium, serum level of iron was also not significantly affected by the studied short period of lead exposure. Iron is an essential trace element which plays an important role in the synthesis of metalloproteins, such as hemoglobin or myoglobin. In an experimental study on rats, a significant reduction in the concentration of serum iron following short- and long-term lead administrations to rats was reported. Authors of this study postulate that lead in the plasma binds to transferrin molecules displacing iron. As a result, unbound iron might be excreted through the kidneys [25]. Besides, an inverse relation between blood lead levels and iron status was reported in children environmentally exposed to lead diagnosed with anemia [26, 27]. This association between the levels of lead and iron is likely due to their sharing of the same transporters, such as the divalent metal transporter 1 (DMT1) and ferroportin 1 (FP1). Consequently, iron status may over- or underregulate the intestinal absorption of lead [28]. However, workers are exposed to lead primarily through the respiratory tract and upregulation of intestinal lead absorption by decreased body iron content should have marginal significance in occupational exposure. Consistently, in our previous study, we did not show any association between blood lead level and serum iron level in a group of chronically lead-exposed workers [6].

The inverse association between lead body burden and zinc status has been also found. These metals also compete in binding to metal transporters, including metallothionein-like transport protein responsible for the metals' intestinal absorption [29]. In the present study, serum zinc level significantly increased after lead exposure compared to baseline. This observation confirms that intestinal absorption of lead in occupational exposure has limited significance. Zinc is essential for cellular metabolism as a central part of over 300 enzymes and proteins [6]. Therefore, as in the case of calcium and magnesium, observed elevation of zinc level may be due to its displacement from protein binding sites in cells. By contrast, the level of plasma copper significantly decreased after a short-term exposure to lead compared to baseline. At the same time, the level of ceruloplasmin, an acute-phase protein that binds copper, did not change significantly. Lead competes with copper analogically to zinc [30]. Therefore, there must exist an additional mechanism that decreases copper level due to the lead action independent of ceruloplasmin metabolism and function. Interestingly, we found a significantly elevated level of copper and ceruloplasmin in chronically lead-exposed workers [6]. This increase in the copper level might have been secondary to the increase of ceruloplasmin level related to the inflammatory processes to be triggered by lead. In light of this, a short-time exposure seems to be not enough to induce such proinflammatory conditions.

Both zinc and copper are necessary for the activity of cytoplasmic and extracellular isoenzymes of SOD, while iron is a cofactor for CAT. SOD utilizes superoxide anions to hydrogen peroxide which is degraded by CAT. Both enzymes serve as a part of the antioxidant defense mechanism against lead-induced oxidative stress [31]. Lead is able to modify expression and activities of CAT and SOD via many mechanisms. On the one hand, lead may induce expressions and activities of CAT and SOD through increased generation of their substrates. On the other hand, activities of CAT and SOD may decrease as a result of lead's binding to thiol groups of their active sites or lead's interactions with their cofactors. Consequently, activities of SOD and CAT in lead-exposed individuals are determined by the sum of opposite mechanisms. This explains divergent results of animal and human studies in this field [5]. In the present study, activities of both enzymes did not change after exposure to lead compared to baseline. However, there were negative correlations between the change of blood level and changes of the activities of leukocyte SOD and Mn-SOD due to lead exposure. The analysis of confounders provided some additional information. In a group of workers, who had a history of occupational exposure to lead, the activities of CAT and SOD in leukocytes were elevated due to the short-term exposure to lead, while in workers without such a history, activities of these enzymes were decreased. These observations suggest an existence of a defense mechanism against lead toxicity that had developed during previous episodes of exposure. The confounding role of smoking habits, age, and BMI also should not be neglected. In a group of nonsmokers, the leukocyte CuZn-SOD activity increased due to the studied lead exposure, while in smokers, the activity of this isoenzyme slightly decreased. Conversely, smokers had significantly higher baseline CAT activity than nonsmokers. The baseline leukocyte activities of CAT were also significantly higher in younger workers and those with lower BMI compared to the group of older workers and those with higher BMI. However, the changes of CAT activities in those groups due to the studied lead exposure were not significantly different. Surprisingly, the activities of SOD and Mn-SOD in leukocytes decreased after the studied lead exposure period in a group of workers with lower BMI, while an opposite trend was observed in those with higher BMI. However, these differences in SOD and Mn-SOD activities were not associated with differences in oxidative stress parameters. Therefore, their significance is limited.

The activities of antioxidant enzymes are associated with oxidative stress intensity. We showed inverse correlations between the changes of leukocyte SOD and CuZn-SOD activities and the changes of MDA and LPH levels due to the studied lead exposure. The ability of lead to induce oxidative stress via impairment of the antioxidant defenses has been widely studied in humans chronically exposed to lead [5, 32]. In the present study, a short-term exposure to lead resulted in increased LPH level by 46%. The levels of serum MDA and LPS did not change significantly. Consistently, in our previous study [8], we showed increased total oxidant status (TOS) level in leukocytes, unchanged leukocyte and erythrocyte MDA level, and decreased erythrocyte glutathione pool due to a short-term lead exposure. Therefore, it seems that the primary products of lipid peroxidation, such as LPH or TOS value, are better in estimating oxidative stress intensity in acute or subacute lead exposure. Additively, the analysis of confounders showed that older workers had greater levels of LPH at baseline and were more susceptible to develop oxidative stress due to lead exposure than the younger population. The possible influence of smoking habits and BMI on measured parameters of oxidative stress was not shown. Surprisingly, subacute exposure to lead resulted in significantly decreased level of LPS in erythrocytes. This parameter serves as a marker of ageing. Lead has been shown to induce eryptosis. In this process, old and damaged erythrocytes are removed from circulation [33]. Therefore, the level of erythrocyte LPS may decrease after exposure to lead due to the elevated removal of the pool of erythrocytes with the highest levels of LPS.

## 5. Conclusions

The plasma and serum levels of essential metals are modified by a short-term exposure to lead in occupationally exposed workers. These modifications are partially different from those observed in a long-term exposure.

A short-term exposure to lead induces oxidative stress associated with elevated levels of LPH. A greater susceptibility to develop oxidative stress due to such exposure is related positively to a higher age of workers. This susceptibility may be a result of an age-related decrease in the CAT activity.

Activities of antioxidant enzymes, such as CAT and SOD, were not significantly affected by a short-term exposure to lead in all examined individuals. However, workers with a history of occupational exposure to lead may develop defensive mechanisms against lead-induced oxidative stress resulting in increased activities of CAT and SOD due to the subsequent episode of exposure.

## Figures and Tables

**Table 1:** Epidemiological data and blood lead levels (B-Pb) in the study population.

	Mean	SD
Lead exposure duration (days)	40	3.2
Age (years)	40.94	13.68
BMI (kg/m^2^)	25.86	3.71
Percentage of smokers (%)	69%	—
B-Pb _before exposure_ (*μ*d/dl)	10.7	7.67
B-Pb _after exposure_ (*μ*d/dl)	49.1	14.1

**Table 2:** The levels of essential metals in the examined population.

	Before exposure		After exposure		Relative change (%)	*p* value
	Mean	SD	Mean	SD		
Ca (mg/dl)	112.55	6.13	116.29	8.34	3	0.042
Mg (mg/dl)	2.09	0.19	2.14	0.19	3	0.045
Se (*μ*g/dl)	69.94	9.55	74.59	9.83	7	0.060
Fe (*μ*g/dl)	156.42	60.33	172.34	64.73	10	0.096
Zn (*μ*g/dl)	92.40	9.02	99.58	15.48	8	0.008
Cu (*μ*g/dl)	95.27	15.07	88.62	11.45	−7	0.028

**Table 3:** The activities of antioxidant enzymes, the levels of oxidative stress markers and ceruloplasmin. S: serum; E: erythrocyte; L: leukocyte.

	Before exposure		After exposure		Relative change (%)	*p* value
	Mean	SD	Mean	SD		
S-SOD (NU/ml)	16.84	2.47	17.42	2.33	3	0.238
E-SOD (NU/mg Hb)	178.69	20.18	175.77	24.23	−2	0.535
L-SOD (NU/mg P)	16.80	1.94	16.89	3.97	1	0.894
L-CuZn-SOD (NU/mg P)	11.14	2.08	11.76	3.35	6	0.373
L-Mn-SOD (NU/mg P)	5.66	1.86	5.21	3.50	−8	0.487
E-CAT (kU/g Hb)	371.27	72.38	393.73	81.89	6	0.200
L-CAT (U/mg P)	38.18	10.61	43.66	14.51	14	0.078
S-MDA (*μ*mol/l)	2.12	0.57	2.37	0.72	12	0.097
S-LPH (*μ*mol/l)	4.72	3.19	6.90	4.66	46	0.005
S-LPS (RF/ml)	1076.05	152.85	1140.59	172.47	6	0.107
E-LPS (RF/g Hb)	2354.00	538.19	2037.71	482.85	−13	0.010
S-CER (mg/dl)	39.43	5.95	40.62	6.83	3	0.324

**Table 4:** Correlations between lead-induced changes of the levels of lead, MDA, and LPH and the changes of the levels of essential metals, activities of antioxidant enzymes, and the levels of oxidative stress markers and ceruloplasmin. S: serum; E: erythrocyte; L: leukocyte; D: a difference between the levels at baseline and at the end of the study period.

	B-Pb-D (*μ*g/dl)	S-MDA-D (*μ*mol/l)	S-LPH-D (*μ*mol/l)
Ca-D (mg/dl)	0.18	0.18	0.25
Mg-D (mg/dl)	0.27	0.14	0.08
Se-D (*μ*g/dl)	0.11	−0.09	0.11
Fe-D (*μ*g/dl)	0.04	0.27	−0.07
Zn-D (*μ*g/dl)	0.07	−0.33	−0.29
Cu-D (*μ*g/dl)	0.17	−0.24	−0.24
S-SOD-D (NU/ml)	−0.08	−0.11	−0.05
E-SOD-D (NU/mg Hb)	0.13	0.15	0.01
L-SOD-D (NU/mg P)	−0.58	−0.29	−0.46
L-CuZn-SOD-D (NU/mg P)	−0.15	−0.34	−0.37
L-Mn-SOD-D (NU/mg P)	−0.55	0.07	−0.07
E-CAT-D (kU/g Hb)	−0.18	−0.08	−0.04
L-CAT-D (U/mg P)	0.01	−0.04	−0.15
S-MDA-D (*μ*mol/l)	0.03	1.00	0.78
S-LPH-D (*μ*mol/l)	0.14	0.78	1.00
E-LPS-D (RF/g Hb)	0.18	0.05	0.14
S-LPS-D (RF/ml)	0.03	0.47	0.42
S-CER-D (mg/dl)	0.07	0.24	0.17

**Table 5:** The comparison between subgroups of workers without a history of occupational exposure to lead (N-HOE) and workers witch such a history (HOE). B: whole blood; S: serum; L: leukocyte; D: a difference between the levels at baseline (1) and at the end of the study period (2).

	N-HOE *n* = 18		HOE *n* = 18		*p value*
	Mean	SD	Mean	SD	
Age (years)	37.72	14.17	44.58	12.37	0.125
Percentage of smokers (%)	0.72	0.46	0.63	0.50	0.569
BMI (kg/m^2^)	25.35	3.81	26.13	3.68	0.530
B-Pb-1 (*μ*g/dl)	4.16	1.62	16.95	5.32	<0.001
B-Pb-2 (*μ*g/dl)	46.52	10.98	51.26	16.37	0.311
B-Pb-D (*μ*g/dl)	42.36	11.04	34.33	16.89	0.028
Se-1 (*μ*g/dl)	68.64	9.25	71.23	9.93	0.424
Se-2 (*μ*g/dl)	78.45	9.70	70.73	8.56	0.016
Se-D (*μ*g/dl)	9.81	14.25	−0.51	12.85	0.029
L-SOD-1 (NU/mg P)	17.34	1.88	16.25	1.89	0.091
L-SOD-2 (NU/mg P)	16.00	4.56	17.79	3.15	0.180
L-SOD-D (NU/mg P)	−1.35	4.03	1.53	3.98	0.038
L-CAT-1 (U/mg P)	39.13	11.08	37.24	10.35	0.600
L-CAT-2 (U/mg P)	38.46	8.84	48.87	17.26	0.029
L-CAT-D (U/mg P)	−0.67	9.89	11.63	22.27	0.039
S-LPH-1 (*μ*mol/l)	3.52	2.12	5.91	3.66	0.022
S-LPH-2 (*μ*mol/l)	6.53	4.76	7.27	4.67	0.644
S-LPH-D (*μ*mol/l)	3.01	3.96	1.36	5.52	0.309

**Table 6:** The comparison between subgroups of smokers and nonsmokers. B: whole blood; E: erythrocyte; L: leukocyte; D: a difference between the levels at baseline (1) and at the end of the study period (2).

	Nonsmokers *n* = 11		Smokers *n* = 25		*p value*
	Mean	SD	Mean	SD	
Age (years)	40.09	13.62	41.40	13.93	0.795
BMI	27.42	3.51	25.17	3.65	0.094
B-Pb-1 (*μ*g/dl)	10.42	7.18	10.82	8.03	0.886
B-Pb-2 (*μ*g/dl)	49.33	10.94	49.04	15.60	0.956
B-Pb-D (*μ*g/dl)	38.91	12.25	38.23	16.13	0.902
Ca-1 (mg/dl)	110.48	5.85	113.46	6.14	0.184
Ca-2 (mg/dl)	120.04	4.89	114.65	9.07	0.018
Ca-D (mg/dl)	9.55	8.62	1.19	10.56	0.027
E-SOD-1 (NU/mg Hb)	168.74	18.76	183.07	19.55	0.048
E-SOD-2 (NU/mg Hb)	174.51	29.20	176.31	22.37	0.841
E-SOD-D (NU/mg Hb)	5.77	25.72	−6.76	28.63	0.221
L-CuZn-SOD-1 (NU/mg P)	10.44	2.15	11.45	2.01	0.186
L-CuZn-SOD-2 (NU/mg P)	13.24	2.23	11.11	3.59	0.078
L-CuZn-SOD-D (NU/mg P)	2.80	3.80	−0.34	3.98	0.034
L-CAT-1 (U/mg P)	35.78	8.49	39.24	11.41	0.374
L-CAT-2 (U/mg P)	37.14	7.78	46.53	15.93	0.023
L-CAT-D (U/mg P)	1.36	12.38	7.29	20.06	0.373

**Table 7:** The comparison between the subgroups of younger and older workers based on the median of age (37 years). B: whole blood; S: serum; L: leukocyte; D: a difference between the levels at baseline (1) and at the end of the study period (2).

	Younger workers *n* = 19		Older workers *n* = 17		*p value*
	Mean	SD	Mean	SD	
Age (years)	29.47	5.25	53.67	6.39	<0.001
Percentage of smokers (%)	0.74	0.45	0.61	0.50	0.428
BMI (kg/m^2^)	24.92	3.70	26.63	3.62	0.164
B-Pb-1 (*μ*g/dl)	8.61	7.18	12.97	7.53	0.080
B-Pb-2 (*μ*g/dl)	48.24	15.08	49.71	13.21	0.756
B-Pb-D (*μ*g/dl)	39.63	15.52	36.76	14.12	0.561
L-CAT-1 (U/mg P)	41.16	10.22	34.85	10.31	0.046
L-CAT-2 (U/mg P)	46.43	16.44	40.57	11.72	0.232
L-CAT-D (U/mg P)	5.27	20.38	5.72	15.77	0.942
S-MDA-1 (*μ*mol/l)	1.98	0.43	2.29	0.68	0.104
S-MDA-2 (*μ*mol/l)	2.04	0.49	2.73	0.78	0.003
S-MDA-D (*μ*mol/l)	0.07	0.55	0.45	1.10	0.193
S-LPH-1 (*μ*mol/l)	3.35	2.72	6.24	3.04	0.005
S-LPH-2 (*μ*mol/l)	5.17	3.54	8.84	5.09	0.016
S-LPH-D (*μ*mol/l)	1.81	3.68	2.60	5.91	0.633
S-LPS-1 (RF/ml)	1113.71	186.24	1033.95	92.30	0.119
S-LPS-2 (RF/ml)	1075.00	157.34	1213.90	162.47	0.014
S-LPS-D (RF/ml)	−38.71	226.46	179.95	187.42	0.004

**Table 8:** The comparison between subgroups of workers with lower and higher BMI based on the median of BMI (25.6 kg/m^2^). B: whole blood; L: leukocyte; D: a difference between the levels at baseline (1) and at the end of the study period (2).

	Low BMI *n* = 17		High BMI *n* = 19		*p value*
	Mean	SD	Mean	SD	
Age (years)	37.44	14.16	44.84	12.22	0.097
Percentage of smokers (%)	0.72	0.46	0.63	0.50	0.569
BMI (kg/m^2^)	22.65	1.96	28.69	2.28	<0.001
B-Pb-1 (*μ*g/dl)	9.84	7.56	11.57	7.69	0.494
B-Pb-2 (*μ*g/dl)	50.68	15.57	47.32	12.59	0.475
B-Pb-D (*μ*g/dl)	40.84	13.76	35.77	15.53	0.302
Se-1 (*μ*g/dl)	66.88	10.40	72.67	8.03	0.069
Se-2 (*μ*g/dl)	77.45	9.72	72.03	9.44	0.099
Se-D (*μ*g/dl)	10.56	13.77	−0.64	13.03	0.017
L-SOD-1 (NU/mg P)	17.21	1.52	16.43	2.22	0.237
L-SOD-2 (NU/mg P)	15.08	4.39	18.52	2.76	0.007
L-SOD-D (NU/mg P)	−2.13	3.42	2.09	3.90	0.002
L-Mn-SOD-1 (NU/mg P)	5.61	1.84	5.70	1.92	0.889
L-Mn-SOD-2 (NU/mg P)	3.83	1.59	6.44	4.27	0.024
L-Mn-SOD-D (NU/mg P)	−1.78	2.12	0.74	4.68	0.049
L-CAT-1 (U/mg P)	42.16	10.35	34.62	9.76	0.031
L-CAT-2 (U/mg P)	45.12	10.16	42.36	17.72	0.576
L-CAT-D (U/mg P)	2.96	13.84	7.73	21.33	0.437
